# Inhibitory Effect of Serotonin Antagonist on Leukocyte-Endothelial Interactions *In Vivo* and *In Vitro*

**DOI:** 10.1371/journal.pone.0147929

**Published:** 2016-01-29

**Authors:** Hiroshi Kataoka, Yuno Ariyama, Michiyo Deushi, Mizuko Osaka, Kosaku Nitta, Masayuki Yoshida

**Affiliations:** 1 Department of Medicine, Kidney Center, Tokyo Women’s Medical University, Tokyo, Japan; 2 Department of Life Sciences and Bioethics, Graduate School of Medical and Dental Sciences, Tokyo Medical and Dental University, Tokyo, Japan; University of Sassari, ITALY

## Abstract

**Background:**

Although 5-HT_2A_ serotonergic antagonists have been used to treat vascular disease in patients with diabetes mellitus or obesity, their effects on leukocyte-endothelial interactions have not been fully investigated. In this study, we assessed the effects of sarpogrelate hydrochloride (SRPO), a 5-HT_2A_ receptor inverse agonist, on leukocyte-endothelial cell interactions in obesity both *in vivo* and *in vitro*.

**Methods and Findings:**

In the *in vivo* experiment, C57BL/6 mice were fed a high-fat high-fructose diet (HFFD), comprising 20% fat and 30% fructose, with or without intraperitoneal injection of 5 mg/kg/day SRPO for 4 weeks. The body weight, visceral fat weight, and serum monocyte chemoattractant protein-1 levels in the mice increased significantly with the HFFD, but these effects were prevented by chronic injections of SRPO. Intravital microscopy of the femoral artery detected significant leukocyte-endothelial interactions after treatment with HFFD, but these leukocyte-endothelial interactions were reduced in the mice injected with SRPO. In the *in vitro* experiment, pre-incubation of activated human umbilical vein endothelial cells (HUVECs) with platelet-rich plasma (PRP) induced THP-1 cell adhesion under physiological flow conditions, but the adhesion was reduced by pretreatment of PRP with SRPO. A fluorescent immunobinding assay showed that PRP induced significant upregulation of E-selectin in HUVECs, but this upregulation was reduced by pretreatment of PRP with SRPO. In other *in vitro* conditions, pre-incubation of THP-1 cells with phorbol 12-myristate 13-acetate increased the adhesion of THP-1 cells to activated HUVECs under rotational conditions, but this adhesion was reduced by pretreatment with SRPO. Western blotting analysis showed that protein kinase C α activation in THP-1 cells was inhibited by SRPO.

**Conclusion:**

Our findings indicated that SRPO inhibits vascular inflammation in obesity via inactivation of platelets and leukocytes, and improvement of obese.

## Introduction

Serotonin/5-hydroxytryptamine (5-HT) and its receptor 5-HT_2A_R, a member of the G protein—coupled receptor (GPCR) family, are known to have effects on atherosclerosis-associated conditions, including body weight [1], abdominal fat weight [2], and glucose and lipid metabolism [3]. The 5-HT_2A_R signaling pathway includes diacylglycerol, protein kinase C (PKC), mitogen-activated protein kinase (MAPK), AP-1, and NF-κB [4–7], which are associated with chronic inflammation in adipose tissue and they are thought to play pivotal roles in the development of atherosclerosis [8]. The 5-HT-dependent pathway plays a role in inflammatory cytokine production [5, 9], leukocyte activation [10], and platelet activation [11]. In particular, activated platelets, which are often found in obese individuals, induce the recruitment of leukocytes to endothelial cells [12]. Activated platelets amplify inflammatory processes through their interactions with vascular cells, blood cells, and cytokines [13].

Sarpogrelate hydrochloride (SRPO), an anti-platelet drug that has been used to prevent thrombosis in atherosclerotic diseases, is a 5-HT_2A_ receptor antagonist. A previous study showed that SRPO decreases the plasma plasminogen activator inhibitor activity [14], as well as reduces the plasma levels of monocyte chemoattractant protein-1 (MCP-1) [15] and soluble E-selectin [16]. In the present study, we assessed the anti-inflammatory effects of SRPO on leukocyte-endothelial cell interactions both *in vitro* and *in vivo*.

## Materials and Methods

### Reagents

The reagents and antibodies used in this study comprised phorbol 12-myristate 13-acetate (PMA) from Wako Pure Chemicals (Tokyo, Japan), RPMI 1640 medium and Dulbecco's phosphate-buffered saline (PBS) from Sigma-Aldrich (St Louis, MO), and anti-PKC α from Santa Cruz Biotechnology, Inc. (Santa Cruz, CA). Western blotting was performed using the standard protocol and ECL reagents (Amersham Biosciences). SRPO was kindly provided by Mitsubishi Tanabe Pharma Corp, Yokohama, Japan.

### Animals

Male C57BL/6 mice (7 weeks old) were obtained from Oriental Yeast (Tokyo, Japan) and fed normal chow (NC) (Clea Japan, Inc., Japan) or a high-fat diet (20% fat, 1.25% cholesterol; Clea Japan, Inc., Japan) with 30% fructose in the drinking water (HFFD). 5 mg/kg/day SRPO diluted with purified water was administered by daily intraperitoneal injection for 4weeks at the same time as the HFFD [17]. The animals were given free access to both food and water. No mortality was associated with these treatments. Following the intravital microscopy (IVM) examination, the heart, thymus, liver, kidney, spleen, and visceral fat were surgically removed and weighed separately. This study was carried out in strict accordance with the recommendations in the Guide for the Care and Use of Laboratory Animals of the National Institutes of Health. The protocol was approved by the Committee on the Ethics of Animal Experiments of Tokyo Medical and Dental University (Permit Number: 0150026A). All surgery was performed under sodium pentobarbital anesthesia, and all efforts were made to minimize suffering.

### Intravital microscopy

IVM examination of femoral arteries was performed after 4 weeks of feeding and intraperitoneal injection of SRPO, as described previously [18]. The mice were anesthetized and ventilated after tracheotomy, where the rectal temperature was kept at 36.0–37.0°C with a heating pad and an infrared heat lamp in order to maintain the body temperature and blood pH. The interaction between rhodamine 6G-labeled leukocytes and the femoral artery was monitored with an epifluorescent microscope (BX51WI, Olympus, Tokyo) and a CCD camera (CoolSnap HQ, Olympus, Tokyo, Japan).

### Quantification of rolling and adherent leukocytes in femoral artery of mice

Leukocyte-endothelial interactions were clearly visualized on the anterior half of the vessel portion facing the objective. All movies were recorded with 5 frames per second. All images were analyzed by using an image analysis software program (MetaMorph; Molecular Devices, Sunnyvale, CA, USA) in accordance with the manufacturer's protocol as previously described [18]. The number of adherent leukocytes that did not move for ≥3 s during the 1-min recording period was counted in a region of interest, i.e., a 100 x 100 μm rectangular segment of the vessel. The number of rolling cells was determined by counting fluorescent cells that passed a reference line perpendicular to the vessel axis.

### Serum MCP-1 level quantification

Blood samples were obtained after the IVM experiment. The serum MCP-1 concentrations were measured using a sandwich enzyme-linked immunosorbent assay, in accordance with the manufacturer's protocol (R&D). An automated microplate reader was used to measure the optical density at a wavelength of 450 nm.

### Cell culture

THP-1, a human monocytic cell line, was obtained from the American Type Culture Collection (Manassas, VA), and the cells were maintained at 37°C in RPMI 1640 medium supplemented with 10% fetal calf serum (FCS), 100 IU/mL penicillin, 100 μg/mL streptomycin, and 2 mmol/L L-glutamine in a humidified 5% carbon dioxide atmosphere. Human umbilical vein endothelial cells (HUVECs) were purchased from Sanko Junyaku (Tokyo, Japan) and cultured in endothelial growth medium-2 (Lonza, Walkersville, MD) at 37°C in a humidified 5% carbon dioxide atmosphere. Plastic culture dishes were precoated with 1% (w/v) collagen and the HUVECs were used for the assays at passages 2–3.

### Platelet-rich plasma preparation

Blood from healthy donors was collected into tubes containing sodium citrate. The samples were centrifuged at 1200 × *g* for 10 min to separate platelet-rich plasma (PRP) from the erythrocytes and leukocytes. The PRP was then transferred to a clean tube and centrifuged at 1200 × *g* for 10 min to separate the platelets from platelet-poor plasma (PPP). PBS was then added to the PRP at a ratio of 1:10.

### Adhesion assay under flow

The protocol for the adhesion assay that mimics physiological flow conditions was described previously [19]. In brief, HUVEC monolayers on coverslips were stimulated with 3 ng/mL TNF-α for 3.5 h, exposed to PPP or PRP for 20 min, and then positioned in a flow chamber mounted on an inverted microscope (Nikon, Tokyo, Japan). PRP was pretreated with or without SRPO (10 μM) [7]. THP-1 cells (1 × 10^6^/mL) were then perfused over the HUVEC monolayers through the chamber with a syringe pump (PHD2000, Harvard Apparatus Inc., Holliston, MA) for 10 min at a controlled flow rate to generate a shear stress of 1.0 dyne/cm^2^. The entire perfusion period was recorded on videotape with a digital video recorder containing a time generator. The captured images were then transferred to a personal computer for image analysis to determine the number of rolling and adherent THP-1 cells on the HUVEC monolayers in 10 randomly selected 15× (magnification) microscope fields (Image Tracker PTV; Digimo, Osaka, Japan).

### Fluorescent immunobinding assay

A FIA was performed as described previously [20]. Briefly, HUVEC monolayers in 96-well plates were stimulated with 3 ng/mL TNF-α for 3.5 h and then exposed to PPP or PRP for 20 min. PRP was pretreated with or without SRPO (10 μM) immediately before addition to the HUVECs. The HUVEC monolayers were then incubated on ice with mouse anti-human E-selectin at a concentration of 10 μg/mL in RPMI plus 1% FCS for 45 min. The wells were washed three times with RPMI plus 1% FCS, and then incubated on ice with an FITC-conjugated goat anti-mouse polyclonal F(ab’)_2_ antibody (Caltag Laboratories) diluted 1:250 in PBS for 45 min. Next, the wells were washed twice with PBS plus 20% FCS, and then twice with PBS alone. The cells were lysed with 0.01% NaOH in 0.1% sodium dodecyl sulfate (SDS) and the fluorescence was measured with a CytoFluor II (Perspective Biosystems) fluorescent plate reader set at 485 nm (excitation)/535 nm (emission), where the results were expressed as relative fluorescent units (RFUs).

### Quantitative leukocyte adhesion assays

The protocol of the non-static rotational adhesion assay has been described previously [21]. THP-1 cells prelabeled with the fluorescent dye BCECF were added (5 × 10^5^ cells/well in RPMI with 1% FCS) to HUVECs monolayers in six-well dishes. After incubation under non-static adhesion assay conditions (rotation at 64 rpm, 10 min, 22–25°C), non-adherent THP-1 cells were removed by washing three times with RPMI plus 1% FCS. The monolayer-associated THP-1 cells were collected into HBSS + 5 mM EDTA + 4 mM EGTA, and their fluorescence was measured using a plate reader. The adhesive interactions of PMA (10 nM, 10 min)-activated THP-1 cells pretreated with or without SRPO (10 μM, 1 h) were monitored using an HUVEC monolayer activated with PMA (10 nM, 8 h). The results were expressed as the percentage of recruited cells, which was calculated as: [(fluorescence retained by recruitment cells)/(fluorescence retained by control cells)] × 100.

### Western blot analysis

To assess the translocation of PKCα, an indicator of activation, from the cytosol to the cell membrane, membrane lysates and total cell lysates of THP-1 cells (1 × 10^6^/mL) were prepared as described previously [19]. An equal amount of protein (10 μg) from each fraction was subjected to 10% SDS—polyacrylamide gel electrophoresis and western blotting analysis was performed with mouse monoclonal antibodies against PKCα (Santa Cruz, CA). The translocation of PKCα was monitored in PMA (10 nM, 10 min)-activated THP-1 cells pretreated with or without SRPO (10 μM, 1 h).

### Statistical analysis

The results were expressed as the mean ± standard error (SE). The data were analyzed by one-way analysis of variance followed by Turkey's post hoc analysis. *P* < 0.05 was considered statistically significant.

## Results

### Chronic effect of sarpogrelate hydrochloride on high-fat high-fructose diet -induced leukocyte-endothelial interactions in the femoral artery *in vivo*

**Fig 1:**
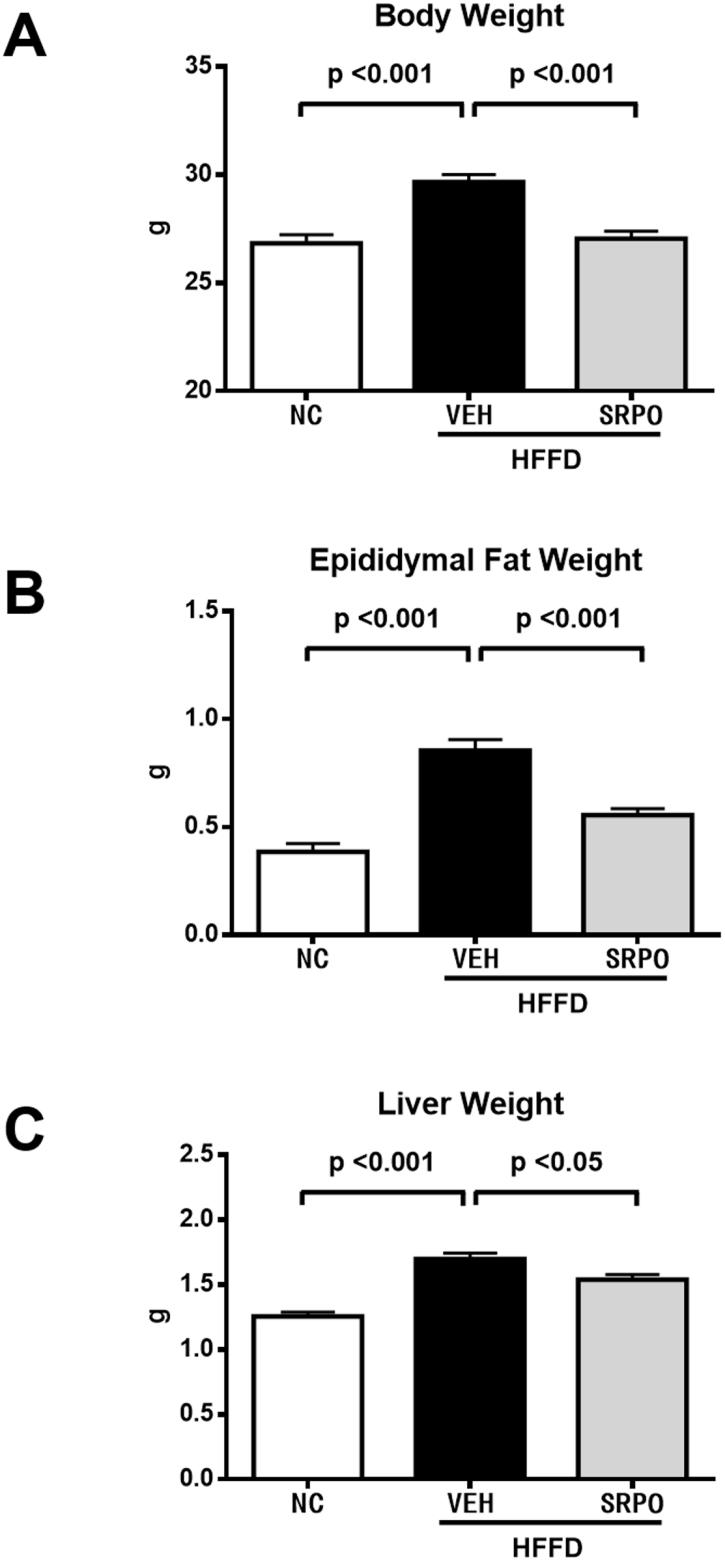
Effects of SRPO on HFFD-induced obesity. (A) Body weight (n = 17, 28, 29). (B) Epididymal fat weight (n = 13, 18, 18). (C) Liver weight (n = 17, 23, 25). Values are the mean ± SE in each group. (Abbreviations: NC = normal chow; HFFD = high-fat high-fructose diet; VEH = vehicle; SRPO = Sarpogrelate hydrochloride.)

First, we examined the effect of SRPO in the HFFD-induced obesity mice model. The characteristics at 11 weeks of age are shown in Fig 1. The body weight, epididymal fat weight, and liver weight were significantly higher in the HFFD with VEH group than those in the NC group, which was blunted in the HFFD with SRPO treatment. There were no significant differences between the groups in terms of the weights of their other organs, including the heart, kidneys, thymus, and spleen.

**Fig 2:**
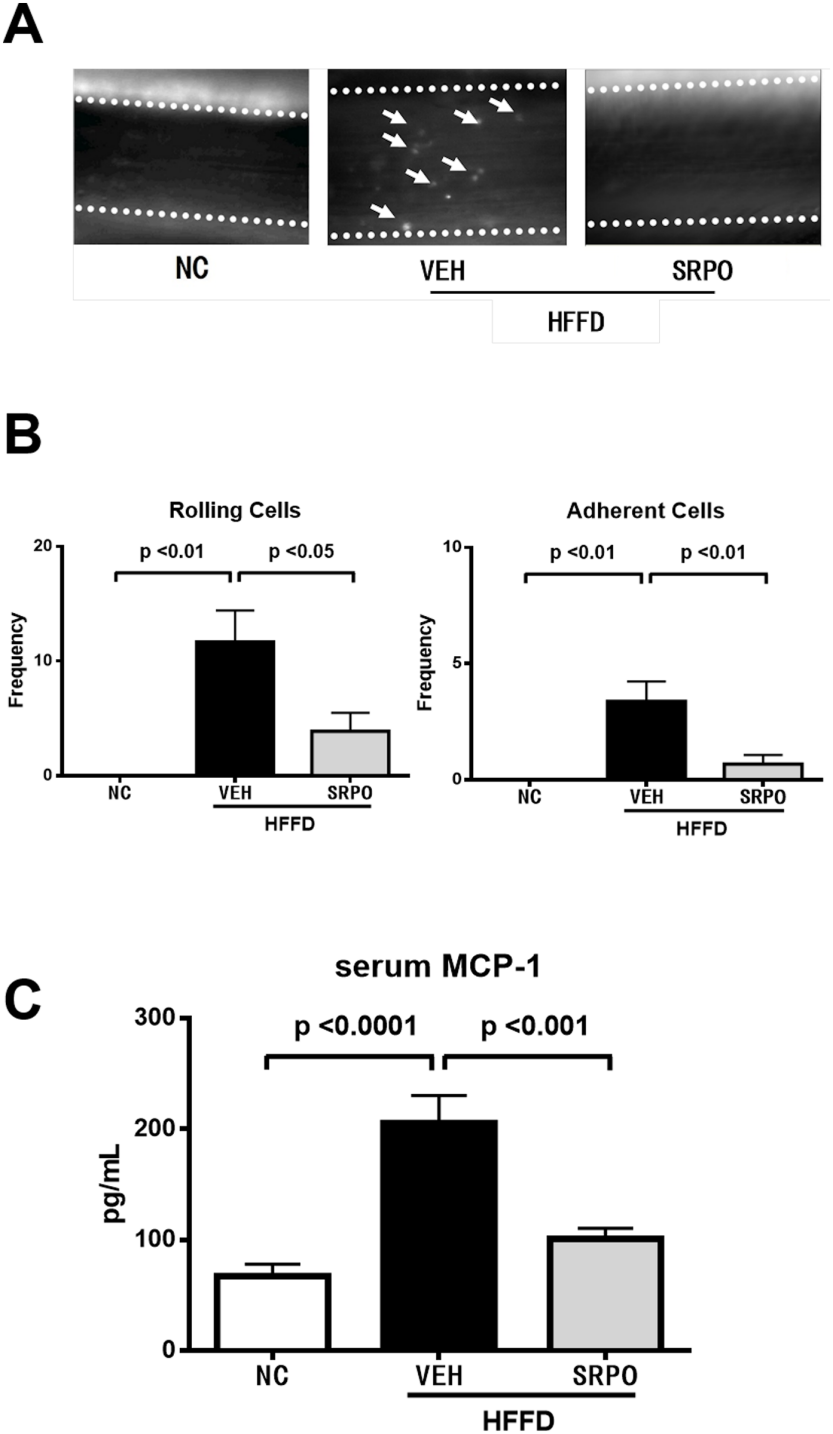
SRPO significantly decreased leukocyte-endothelial interactions and serum MCP-1 level. (A) Image of leukocyte-endothelial interactions in the femoral arteries of mice (with the margins of vessels indicated by dotted lines). Arrows indicate adhered or rolling leukocytes. (B) The numbers of rolling (left) and adherent cells (right) in all groups were calculated as described in Methods. Values are the mean ± SE (n = 10, 16, 18). (C) Effect of SRPO on serum MCP-1 levels in HFFD-induced obesity. Values are the mean ± SE (n = 13, 13, 11). Serum MCP-1 level of the HFFD + VEH group was higher than in the NC group, and SRPO prevented the increase in serum MCP-1 level on the HFFD + VEH group.

We then conducted an IVM analysis to measure the effect of SRPO on HFFD-induced leukocyte-endothelial interactions in the femoral artery (S1, S2 and S3 Videos). As shown in Fig 2A and 2B, HFFD with VEH induced significantly more leukocyte-endothelial interactions compared with the NC group (Rolling cells, *P* < 0.01; Adherent cells, *P* < 0.01), but this was significantly reduced by SRPO (Rolling cells, *P* < 0.05; Adherent cells, *P* < 0.01). Concurrent serum measurements showed that the MCP-1 level was significantly elevated in those fed the HFFD with VEH compared with NC (*P* < 0.001), but this was reduced by SRPO treatment (*P* < 0.001) (Fig 2C).

### Effect of sarpogrelate hydrochloride on platelet-rich plasma-induced leukocyte adhesion to activated human umbilical vein endothelial cells under flow

**Fig 3:**
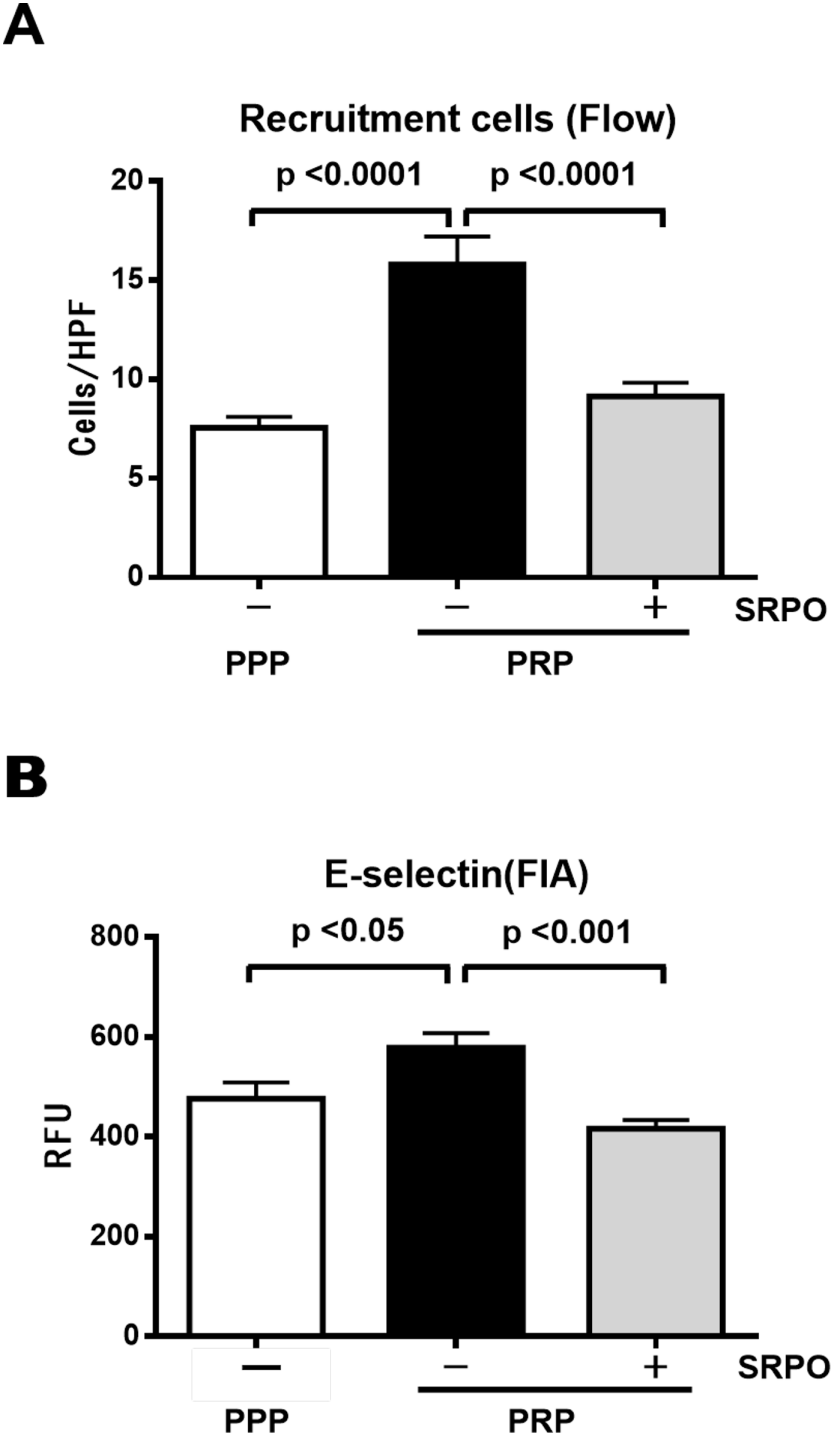
THP-1 cell adhesion to HUVECs (Flow chamber assay) and expression of E-selectin in HUVECs. (A) SRPO significantly reduced the number of PRP-induced THP-1 cell that adhered to HUVECs. HUVEC monolayers were stimulated with 3 ng/ml TNF-α for 3.5 h and exposed to PPP or PRP for 20 min. PRP was pretreated or not with SRPO (10 μM) just before addition to the HUVECs. THP-1 cells were perfused over activated HUVEC monolayers at a flow rate of 1.0 dyne/cm^2^. Adhesion assays were performed as described in Materials and Methods. Data are the mean ± SD of three independent experiments in each group. (B) SRPO significantly reduced the expression of PRP-induced E-selectin in HUVECs. E-selectin expression was determined by FIA as described in Materials and Methods. Data are the mean ± SD of 6 independent experiments in each group.

SRPO was found to modulate platelet activation, so we investigated the effect of SRPO on PRP-induced THP-1-HUVEC interactions under flow conditions *in vitro* (shear stress of 1.0 dyne/cm^2^). As shown in Fig 3A, PRP increased THP-1 cell adhesion to activated HUVECs (PPP, 7.6 ± 0.6/HPF vs. PRP, 15.5 ± 1.4/HPF; *P* < 0.001). Pre-incubation of PRPs with SRPO reduced their adhesion (PRP + SRPO, 9.2 ± 0.7/HPF; *P* < 0.001) (n = 20 in each group).

Cell surface E-selectin expression was upregulated significantly by PRP (PPP, 476.7 ± 32.6 RFU vs. PRP, 578.0 ± 29.6 RFU; *P* < 0.05), but this upregulation was reduced by pre-incubation with SRPO (PRP + SRPO, 416.3 ± 17.4 RFU; *P* < 0.001) (Fig 3B).

### Effect of sarpogrelate hydrochloride on phorbol 12-myristate 13-acetate-induced leukocyte adhesion to activated human umbilical vein endothelial cells *in vitro*

**Fig 4:**
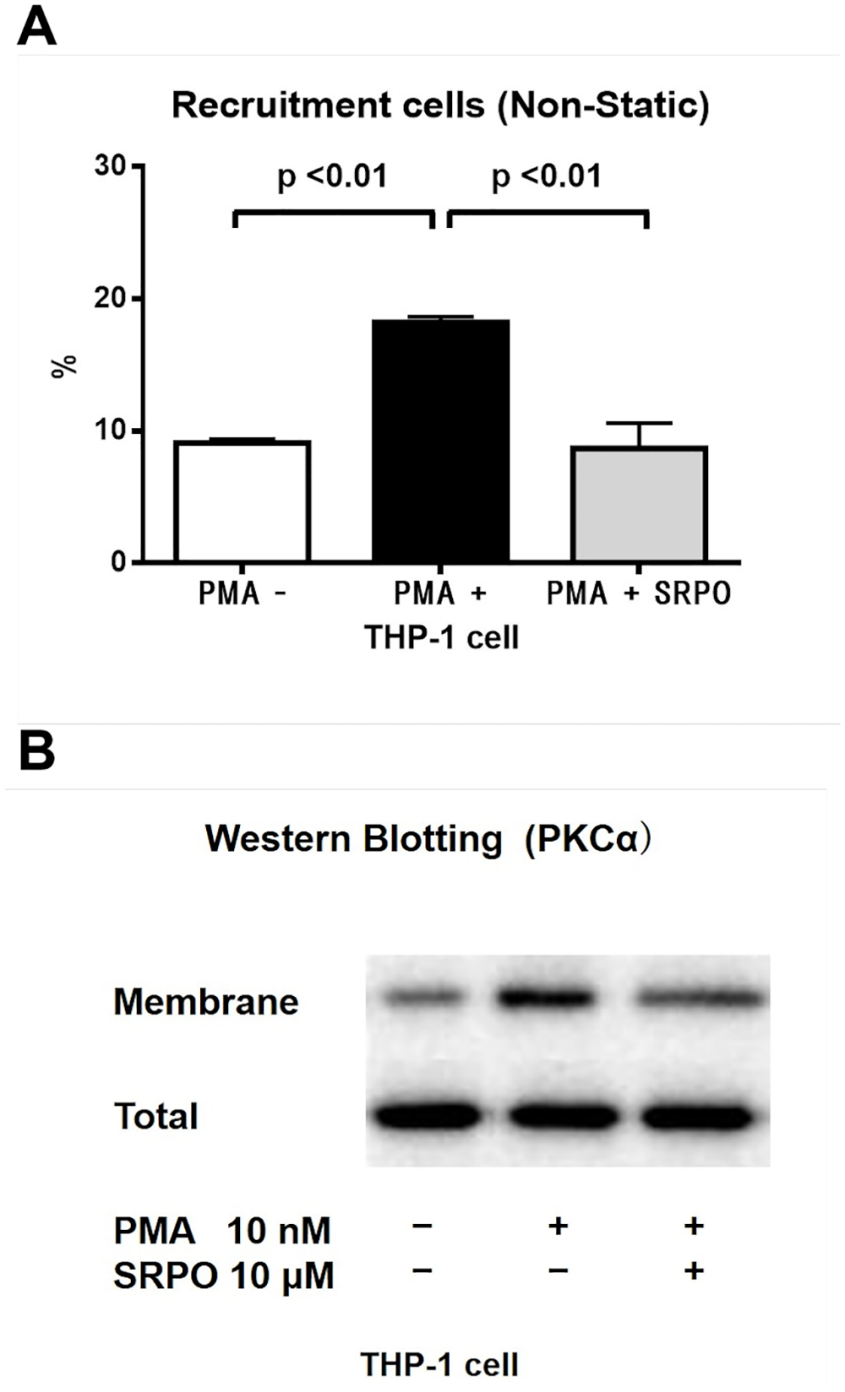
THP-1 cell adhesion to HUVECs (Non-static rotational assay) and expression of PKCα in THP-1 cells. (A) SRPO significantly reduced PMA-triggered THP-1 cell adhesion to the HUVECs. THP-1 cells were pretreated with SRPO (10 μM) for 1 h and stimulated with PMA (10 nM) for 10 min before the assay. Preliminary experiments with trypan blue staining demonstrated that THP-1 cells were not damaged by SRPO (10 μM, 1h) treatment (data not shown). Adhesion assays were performed as described in Materials and Methods. Data are the mean ± SD of three independent experiments in each group. (B) SRPO attenuated PMA-induced PKCα activation in THP-1 cells. THP-1 cells were incubated in the presence or absence of SRPO (10 μM) for 1 h and stimulated with PMA (10 nM) for 10 min before the assay, and membrane proteins and total PKCα protein were detected by immunoblotting. Data are representative of three independent experiments.

To directly assess the molecular mechanisms of the anti-inflammatory effects of SRPO, we conducted a THP-1-HUVEC adhesion assay *in vitro* under non-static rotational conditions. PKCα is important during integrin-mediated cell adhesion [22], so we used PMA, a potent PKC activator, to assess leukocyte adhesion. Treatment of THP-1 cells with PMA (10 nM, 10 min) significantly increased their adhesion to HUVECs under non-static conditions (*P* < 0.001). By contrast, pre-incubation of THP-1 cells with SRPO (10 μM, 1 h) reduced the PMA-induced THP-1 cell adhesion to PMA-activated HUVECs (10 nM, 8 h; *P* < 0.01) (Fig 4A).

In monocytes, PKCα is one of the key modulators of the inflammatory process, including leukocyte-endothelial interactions [22]. To explore the molecular mechanisms of the anti-adhesive action of SRPO, we examined the effect of SRPO on the PKCα activity levels in THP-1 cells. As shown in Fig 4B, the results of the immunoblotting analyses indicated that PMA increased the amount of PKC-α protein in THP-1 cell membrane fractions [PMA (-), 9.1 ± 0.3% vs. PMA (+), 18.2 ± 0.4%; *P* < 0.01], whereas the increase in PKC-α protein was reduced by SRPO (PMA + SRPO, 8.7 ± 1.9%; *P* < 0.01) (n = 3 in each group).

## Discussion

The results of this study demonstrate that the administration of SRPO reduced leukocyte-endothelial cell interactions through its effects on platelets, monocytes, and adipose tissue both *in vitro* and *in vivo*. SRPO is a 5-HT_2A_R antagonist, which is used clinically as an antiplatelet drug, and is not known to antagonize 5-HT_2B_ receptor and others [23]. 5-HT_2A_R is widely expressed in a variety of cells, including platelets [24], monocytes [10, 25], adipocytes [7, 26], and smooth muscle cells [27]. 5-HT2R is a phospholipase C stimulator and a member of the GPCR family. Several studies have shown that the 5-HT_2A_R signaling pathway includes diacylglycerol, PKC, MAPK, AP-1, and NF-κB [4–7], and thus GPCR activation can theoretically affect multiple signaling pathways [28, 29]. However, inverse agonism is a recently discovered feature of GPCR systems [30, 31]. Interestingly, 5-HT_2A_R has also been reported to exhibit constitutive activity, and SRPO has been reported to exhibit a potent inverse agonist activity against 5-HT_2A_R [32]. Based on the GPCR cross-talk theory, an inverse agonist of one GPCR may result in the inhibition of multiple signaling pathways [33]. Therefore, SRPO can act as an antiplatelet drug but also as an inverse agonist of 5-HT_2A_R in a variety of cells, which may explain the pleiotropic atheroprotective effects of SRPO. In this study, we examined the pleiotropic effects of SRPO on leukocyte-endothelial cell interactions both *in vivo* and *in vitro*.

First, we investigated the effect of SRPO on HFFD-induced leukocyte-endothelial interactions in the femoral artery using an IVM technique, which we had developed to directly monitor leukocyte interactions with atheroprone arteries *in vivo* [18]. Chronic administration of SRPO significantly reduced the body weight, visceral fat weight, serum MCP-1 levels, and leukocyte-endothelial cell interactions in the HFFD + VEH group.

It has been proposed that fat mass growth in obesity is a result of adipocyte hypertrophy and the recruitment of new adipocytes from pre-adipocytes [34]. 5-HT_2A_R is expressed in adipocytes [7, 26] and 5-HT has been shown to accelerate adipocyte differentiation, whereas 5-HT_2A_R antagonists reduce adipogenesis [26]. Uchida-Kitajima et al. reported that the expression of 5-HT_2A_R increased during adipocyte differentiation in hypertrophic adipocytes, and the 5-HT_2A_R signaling pathway stimulated MAPK in adipocytes [7]. Other studies have shown that 5-HT_2A_R inhibition induced reductions in body weight [1, 2] and the visceral fat weight [2] in high-fat diet-fed rats. Thus, SRPO may have inhibited adipocyte proliferation and differentiation via the 5HT_2A_R signaling pathway in the HFFD-fed obese mice, thereby resulting in decreased body weight and visceral fat weight (Fig 1).

In obesity, the adipokines secreted by adipose tissue have proinflammatory and atherogenic effects [35]. In particular, MCP-1 is a potent chemokine that is upregulated in obesity and it plays a key role in leukocyte-endothelial cell interactions [22], which comprise the crucial initial step in the pathogenesis of atherosclerosis [36]. Adipose tissue is an important source of MCP-1 [37], and the MCP-1 mRNA and protein levels have been reported to be upregulated in the adipose tissue of high-fat diet-fed obese mice [38]. MCP-1 gene expression is regulated by the MAPK, AP-1, and NF-κB signaling pathways [39]. The MCP-1 secreted by adipocytes adheres to glycosaminoglycans on the surfaces of endothelial cells [40] and it attracts leukocytes to vascular endothelial cells by activating endothelial cells and monocytes. MCP-1 activates THP-1 cells by activating the PKCα signaling pathway, which results in the upregulation of α4 and β2 integrins [22].

The results of the present study indicate that increases in the serum MCP-1 levels in HFFD-fed obese mice were accompanied by increases in the visceral fat volume and in leukocyte-endothelial cell interactions, whereas all of these increases were prevented by SRPO. 5-HT_2A_R is also expressed in adipocytes [7, 26] and monocytes [10, 25]. SRPO may indirectly inhibit MCP-1 production by reducing the adipose tissue volume and directly inhibit MCP-1 production via its inverse agonist action on the 5HT2AR, thereby reducing MAPK, AP-1, and NF-κB activation. Given the inverse effects of SRPO on GPCR signaling cross-talk, SRPO may also reduce the effects of MCP-1 via the 5-HT_2A_R/PKC signaling pathway. Overall, the inverse effects of SRPO on the MAPK/NF-κB or PKC signaling pathways may inhibit both MCP-1 production and the effects of MCP-1, thereby reducing leukocyte-endothelial cell interactions in HFFD-fed obese mice (Fig 2).

SRPO was originally used as an antiplatelet drug, so the influence of antiplatelet effects of SRPO must also be considered. Obesity is associated with increases in platelet activation [41] and the parameters that reflect platelet activation, such as the mean platelet volume, soluble P-selectin, and soluble CD40 ligand (sCD40L), are also increased in the blood of obese patients [42]. Platelet activation is induced by interactions between several agonists and GPCRs that recognize thrombin, adenosine diphosphate, thromboxane A2, platelet activating factor, epinephrine, 5-HT, and chemokines, including MCP-1 [43]. The platelets activated by the GPCR/PKC signaling pathway amplify inflammatory processes through pleiotropic interactions with vascular cells, blood cells, adipocytes, and adipokines. For example, activated platelets induce MCP-1 secretion [44] and the expression of E-selectin on endothelial cells via an NF-κB-dependent signaling pathway [45]; and sCD40L is released by activated platelets to initiate various inflammatory responses [13], including E-selectin expression [46] and the release of chemokines including MCP-1 [46]. Incubation of human adipocytes with recombinant CD40L induces the expression of MCP-1 and adipogenesis via the MAPK/NF-κB signaling pathway [47]. Incubation of monocytes with CD40L activates PKC or the MAPK/NF-κB signaling pathway [48, 49]. 5-HT_2A_R is expressed in platelets [24], monocytes [10, 25], and adipocytes [7, 26]; thus, SRPO may reduce leukocyte-endothelial cell interactions in HFFD-fed obese mice by inhibiting the effects of activated platelets.

We also investigated the detailed molecular mechanisms using an *in vitro* leukocyte adhesion assay system with PPP or PRP. We identified a potential role for endothelial E-selectin in PRP-mediated THP-1 adhesion to HUVECs (Fig 3). E-selectin is a member of the selectin family of transmembrane glycoproteins and it is constitutively expressed on endothelial cells, while it also promotes monocyte rolling and adhesion to the endothelium [19]. It is known that activated platelets induce the expression of E-selectin on endothelial cells [45]. In humans, the soluble E-selectin levels are increased in obesity, whereas they decrease with weight loss [50], and SRPO has been reported to reduce the soluble E-selectin levels [16]. Our data suggest that SRPO at least partly reduces PRP-triggered monocyte adhesion to activated HUVECs by modulating E-selectin upregulation in HUVECs. Moreover, we demonstrated an anti-adhesive effect of SRPO on PMA-activated THP-1 cells (Fig 4). Several lines of evidence indicate that PKCα is important for integrin-mediated cell adhesion. Activation of the PKCα signaling pathway in monocytes, which results in the upregulation of integrins, causes leukocyte-endothelial interactions [22]. The activation of PKCα has been reported to be implicated in the etiology of diabetes or obesity in animal models [51, 52], and SRPO has been reported to inhibit the 5-HT_2A_R/G protein/PKC pathway in monocytes [10]. 5-HT_2A_R is expressed in monocytes [10, 25], so our data suggest that SRPO at least partly reduces PMA-induced monocyte adhesion to activated HUVECs by modulating PKCα activation in THP-1 cells. The potential mechanisms underlying this process appear to involve the inverse agonist activity of SRPO against the 5-HT_2A_R/PKC signaling pathway.

## Conclusion

In summary, SRPO inhibited leukocyte-endothelial interactions enhanced by platelets in vitro. Potential mechanism seems to involve PKCa activation in leukocytes. We demonstrated that SRPO reduced leukocyte adhesion to femoral artery in HFFD-treated mice in vivo. SRPO also reduced visceral fat weight and serum MCP-1 level in these mice. These data indicate novel anti-inflammatory role of SRPO in metabolic syndrome.

## Supporting Information

S1 Video NC (Leukocyte-endothelial Interactions: Intravital microscopy). (MP4)

S2 Video HFFD + VEH (Leukocyte-endothelial Interactions: Intravital microscopy). (MP4)

S3 Video HFFD + SRPO (Leukocyte-endothelial Interactions: Intravital microscopy). (MP4)
